# Influence of Optimization Algorithms and Computational Complexity on Concrete Compressive Strength Prediction Machine Learning Models for Concrete Mix Design

**DOI:** 10.3390/ma18061386

**Published:** 2025-03-20

**Authors:** Patryk Ziolkowski

**Affiliations:** Faculty of Civil and Environmental Engineering, Gdansk University of Technology, Gabriela Narutowicza 11/12, 80-233 Gdansk, Poland; patziolk@pg.edu.pl; Tel.: +48-58-347-2385

**Keywords:** applied machine learning, concrete, concrete mix design, concrete strength prediction, data mining, sustainable, buildings, sustainability, construction industry, innovation, cement, green building, sustainable development

## Abstract

The proper design of concrete mixtures is a critical task in concrete technology, where optimal strength, eco-friendliness, and production efficiency are increasingly demanded. While traditional analytical methods, such as the Three Equations Method, offer foundational approaches to mix design, they often fall short in handling the complexity of modern concrete technology. Machine learning-based models have demonstrated notable efficacy in predicting concrete compressive strength, addressing the limitations of conventional methods. This study builds on previous research by investigating not only the impact of computational complexity on the predictive performance of machine learning models but also the influence of different optimization algorithms. The study evaluates the effectiveness of three optimization techniques: the Quasi-Newton Method (QNM), the Adaptive Moment Estimation (ADAM) algorithm, and Stochastic Gradient Descent (SGD). A total of forty-five deep neural network models of varying computational complexity were trained and tested using a comprehensive database of concrete mix designs and their corresponding compressive strength test results. The findings reveal a significant interaction between optimization algorithms and model complexity in enhancing prediction accuracy. Models utilizing the QNM algorithm outperformed those using the ADAM and SGD in terms of error reduction (SSE, MSE, RMSE, NSE, and ME) and increased coefficient of determination (R^2^). These insights contribute to the development of more accurate and efficient AI-driven methods in concrete mix design, promoting the advancement of concrete technology and the potential for future research in this domain.

## 1. Introduction

Recent advances in concrete mix design have increasingly incorporated machine learning (ML) techniques, which offer promising capabilities for predicting the technical parameters of concrete. These models hold the potential to transform the construction industry by delivering a more accurate and efficient approach to mix design. However, the accuracy and efficiency of these ML models are influenced by a variety of factors, including the computational complexity of the models and the optimization algorithms employed during training. This study aims to extend the understanding of these factors by investigating the combined impact of computational complexity and different optimization algorithms on the predictive performance of ML models for concrete. By exploring the relationship between these elements, the research provides valuable insights into the trade-offs between model accuracy, computational efficiency, and the role of optimization in enhancing prediction outcomes. The findings will guide the development of more efficient and accurate ML models for concrete mix design in the future.

Concrete mix composition typically consists of cement, water, a combination of fine and coarse aggregates, and supplementary materials known as additives and admixtures. These components are precisely formulated to enhance the chemical properties and performance of concrete, particularly in terms of compressive strength, durability, and workability. A wide range of additives and admixtures are employed to improve specific properties of concrete [1,2,3,4], such as accelerators for faster setting times, water-reducing agents, durability enhancers, set-retarding admixtures, and fibres for reinforcement [5,6]. Properly designing a concrete mixture is a crucial aspect of the construction process and requires the consideration of economic, environmental, and performance factors. An optimal mix must balance cost-effectiveness with the ability to meet specific construction and environmental requirements, such as compressive strength, resistance to environmental degradation (e.g., chloride ingress [7,8]), and, increasingly, low carbon emissions. Solutions like graphene nanoparticle admixtures have been proposed to address environmental concerns by reducing carbonation and enhancing concrete durability [9,10]. The hydration process, initiated when water interacts with cement, is fundamental to concrete hardening and strength gains [11]. This process leads to the formation of compounds such as calcium silicate hydrate [12], which bond aggregates together, progressively increasing the strength of the concrete over time. Most concrete achieves a significant portion of its compressive strength within 28 days, though some types may take longer [13]. Traditional methods for concrete mix design, often based on empirical estimations of material strength and derived from techniques developed decades ago, struggle to address the complexities of modern concrete materials and chemical compositions [14,15]. These conventional methods are labour-intensive and often ineffective, highlighting the need for new, technologically advanced solutions.

ML has emerged as a potential solution to these challenges, offering robust tools for predicting the compressive strength of concrete based on various mix design parameters. ML, a subfield of artificial intelligence, enables systems to learn from the data, identify patterns, and make predictions with minimal human intervention [16]. Among the ML paradigms, supervised learning, where models are trained on labelled data, is particularly well-suited to predicting concrete properties. Popular algorithms used in this domain include linear regression [17], decision trees [18,19], support vector machines [20], and neural networks, often fine-tuned through hyperparameter optimization to improve performance. Deep learning, a subset of ML that uses multi-layered neural networks, has proven particularly effective in handling high-dimensional data and complex prediction tasks [21,22]. Neural networks are adept at learning intricate patterns from large datasets, which makes them suitable for predicting material properties like concrete compressive strength. However, the performance of these models is influenced not only by their architecture and computational complexity but also by the optimization algorithms employed during training. In ML, optimization algorithms adjust the model’s parameters to minimize error and improve accuracy. The choice of optimization method can significantly affect model performance and efficiency, yet this aspect has been underexplored in concrete mix design research. This research work used three optimization algorithms: the Quasi-Newton Method (QMN), Adaptive Moment Estimation (ADAM), and Stochastic Gradient Descent (SGD). The QNM is an optimization algorithm that approximates the Hessian matrix to efficiently locate the minimum of the loss function. Unlike traditional gradient descent, it avoids the direct computation of the Hessian, which can be computationally expensive, making it a practical choice for improving convergence speed while maintaining accuracy. ADAM builds on Stochastic Gradient Descent (SGD) by adapting the learning rate for each parameter using estimates of the first and second moments of the gradients. This adaptability often results in faster convergence and robust performance, particularly in scenarios with sparse gradients or noisy data. SGD is a widely used optimization technique that updates model parameters in the direction of the negative gradient based on a subset of the data (mini-batch). By reducing computational demands and aiding the model in avoiding local minima, SGD is well-suited for large-scale machine learning applications.

## 2. AI-Driven Approach in Concrete Mix Design

### 2.1. Optimal Concrete Properties and Traditional Concrete Mix Design

Creating an optimal concrete mix is a highly intricate process that demands a deep understanding of concrete technology along with practical expertise. The primary goal is to identify the correct combination of materials to achieve the desired properties in both fresh and hardened concrete. Throughout the stages of production, ranging from mixing and transporting to curing, various mechanical and chemical characteristics play a critical role. These characteristics include factors such as the plasticity, durability, compressive strength, and modulus of elasticity, all of which impact the concrete’s performance [23]. The significance of each property depends on the specific application; for example, compressive strength is crucial for maintaining structural stability, while durability is paramount in harsh environmental conditions. Inadequately designed concrete mixes can lead to severe consequences, including compromised structural integrity and increased costs. As a result, manufacturers often go beyond the specified design requirements to ensure that the concrete meets or exceeds the necessary performance standards, avoiding the risks associated with non-compliance [24].

Concrete mix design practices vary significantly across different regions, although certain standards are widely shared. In the European Union (EU), the key standard governing concrete technology is EN 206 [25,26], which outlines the requirements for the specification, performance, production, and conformity of concrete [25]. For structural concrete design, Eurocode 2 (EN 1992-1-1 [27]) provides further guidelines. Each EU member state adapts these standards to their national context; for example, Germany follows DIN EN 206 [26], while Poland uses PN-EN 206 + A1: 2016-12 [28]. In Poland, several design methods are commonly employed, such as those developed by Bukowski, Eyman, Klaus, Kopycinski, and Paszkowski, alongside the double-coating technique [29]. By contrast, in the United States, mix design often relies on methods like the Bolomey and Fuller curves [14] or the 0.45 power gradation chart [30,31]. Most of these methods, regardless of regional differences, are derived from the “Three Equations Method”, which integrates both experimental and analytical techniques to optimize concrete mix design. The Three Equations Method traditionally involves a process where the necessary proportions of cement, water, and aggregate are determined by analytical calculations and validated through destructive laboratory testing. This method helps establish the weight of each component per unit volume, accounting for factors like consistency, strength, and impermeability.

(1) $$W=C\cdot w_{c}+K\cdot w_{k}\;[L],$$

(2) $$f_{cm}=A\left[\left(\frac{C}{W}+p\right)-a\right]\;[MPa],$$

(3) $$f_{cm}=A_{1,2}\left(\frac{C}{W}\pm a\right)\;[MPa],$$

(4) $$\frac{C}{\rho_{c}}+\frac{K}{\rho_{k}}+W=1000\;[{dm}^{3}],$$

Equation (1) governs water requirements, helping to determine the optimal consistency of the mix based on aggregate and cement characteristics, while Equations (2) and (3) (the Bolomey and Feret formulas [32]) link compressive strength to the water–cement ratio, cement grade, and aggregate. The final formula, Equation (4), ensures that the total volume of the individual components in the concrete mix equals the total volume of the concrete. While the Three Equations Method has been a useful starting point for mix design, modern concrete technology demands more sophisticated techniques that account for the variability of materials and the growing complexity of additives and admixtures. As a result, traditional methods, which rely on empirical formulas, have limitations in addressing the diverse properties of contemporary concrete [15]. The process of concrete mix design also involves several practical steps, starting with the formulation of initial assumptions and the definition of the desired properties of both fresh and hardened concrete. Important considerations include the location and intended use of the concrete, the degree of reinforcement, and the geometric factors of the structure. The key properties of fresh concrete, such as bulk density, consistency, and air content, must be considered [33,34], while the focus for hardened concrete shifts to factors like frost resistance, fire resistance, and compressive strength. Furthermore, the technological processes involved, such as the curing conditions and compaction methods, are critical for ensuring the desired performance characteristics are achieved. The concrete exposure class [35,36], which determines the environmental stress resistance of the mix, is another vital consideration. Properties like impermeability, maximum aggregate size, and workability need to be determined to ensure a mix design that is both durable and efficient [37,38]. The design process concludes with laboratory tests to verify the mix’s properties, followed by adjustments based on real-world conditions, such as moisture content in the aggregate or transportation logistics.

### 2.2. Predictive Modelling of Concrete Properties Using Machine Learning

ML provides an advanced alternative to conventional concrete mix design methods by leveraging large datasets to predict key technical properties of concrete, such as compressive strength. In recent years, ML models have proven highly effective in this domain, as they can capture complex, nonlinear relationships between mix components and the resulting concrete properties. These models can be used to minimize trial-and-error in the mix design process and reduce over-engineering by accurately predicting outcomes based on the data. By integrating ML into the mix design process, concrete producers can optimize raw material usage, improve performance predictions, and reduce environmental impact. The success of ML models in predicting concrete properties depends on several factors, including the complexity of the model and the optimization algorithm used during training.

The initial use of ML to predict concrete strength was introduced by Yeh et al. [39] in 1998. They employed artificial neural networks (ANNs) and linear regression to predict the strength of high-performance concrete using seven input variables. However, their dataset included samples at the early stages of maturity, as young as three days old, which may have skewed the results due to the incomplete curing process. This limitation highlighted the need for more refined approaches to model concrete compressive strength. In 2003, Seung-Chang Lee [40] refined this concept by introducing a modular ANN architecture with five networks, each corresponding to different stages of concrete maturation. Although his method offered a unique way to model strength development over time, its practical relevance was questioned since engineers are primarily concerned with concrete that has reached full or near-full strength. His use of parameter condensation to determine the optimal number of neurons improved network performance but had limited application for real-world scenarios focused on final strength prediction. Hola J. and Schabowicz K. [41,42] proposed a non-destructive approach to predicting concrete compressive strength in 2005, bypassing the reliance on mix composition. Instead, they trained an ANN model using data from non-destructive testing tools, such as ultrasonic velocity and pull-out strength measurements. Their model was trained using the Levenberg–Marquardt algorithm and incorporated eight hidden neurons. They demonstrated that the predictions from their model were comparable to traditional non-destructive test results. However, the complexity of using non-destructive testing tools added practical challenges for their widespread application. In 2006, Gupta et al. [43] introduced a neural-expert system that used interval training patterns for predicting the strength of high-performance concrete. Although their system employed a generalized backpropagation algorithm, the inclusion of unrelated variables such as curing time complicated the model. This approach risked overfitting on irrelevant patterns, which limited its generalizability. With the rise of deep learning, Fangming Deng et al. [44] in 2018 shifted the focus to using deep neural networks (DNNs) for concrete strength prediction. They trained their model on recycled concrete samples, leveraging deep feature extraction to improve generalization. Their research demonstrated that DNNs provided better overall accuracy compared to traditional ANNs, though their limited dataset of 74 samples risked underfitting the model. Despite this, their work laid the groundwork for further exploration into deep learning’s potential for concrete properties prediction. A 2019 study by Ziolkowski P. et al. [45] developed an algorithm specifically for designing concrete-based mixes. While this algorithm accurately predicted compressive strength for lower-strength concrete, it struggled with high-strength mixes above 40 MPa and had difficulty incorporating the effects of additives and admixtures, a critical aspect in modern concrete technology. More recent studies have explored ML’s potential for predicting properties of sustainable concrete mixtures. In 2020, Nunez I. and his team [46] investigated the prediction of compressive strength for recycled aggregate concrete. They developed three ML models based on Gaussian processes, recurrent neural networks, and gradient-boosted regression trees and demonstrated that gradient boosting achieved the highest accuracy. This highlighted the importance of selecting the right optimization method when using ML to predict concrete properties. Further expanding on the role of optimization algorithms, Marani A. et al. [47] explored the use of generative adversarial networks (GANs) in their 2020 study on ultra-high-performance concrete. By augmenting their dataset with synthetic data, they significantly improved the performance of their ML model, showcasing how data augmentation techniques and advanced optimization methods can enhance prediction accuracy in scenarios with limited real-world data. In 2021, Ziolkowski P. et al. [48] introduced an adaptive ML method that improved the estimation of concrete compressive strength. This adaptive model considered variability within concrete mixes more effectively than previous static models, providing more accurate predictions. The authors demonstrated how modern ML models, when properly optimized, can surpass traditional methods in both accuracy and efficiency. In parallel, Adil M. et al. [49] examined how varying the number of neurons and layers in an ANN impacts the accuracy of general concrete mix design predictions. Their work confirmed that optimizing the number of hidden layers and neurons is essential to improving prediction performance, reinforcing the idea that hyperparameter optimization is critical for refining ML models in concrete mix design. Feng W. et al. [50] explored the mechanical properties of rubber-modified recycled aggregate concrete (RRAC) using ML techniques. Their research employed a beetle antennae search (BAS) algorithm for hyperparameter tuning, ultimately finding that backpropagation neural networks (BPNN) provided the best predictions of compressive strength and peak strain. This study further emphasized the importance of optimizing ML models to improve the prediction of sustainable concrete properties. Studies by Tavares C. et al. [51,52] and Endzhievskaya I.G. et al. [53] contributed additional insights into the use of ensemble ML techniques, such as random forests and decision trees, for optimizing ultra-high-performance concrete (UHPC) mix design and road concrete, respectively. Both studies demonstrated that by integrating optimization methods with ML, it is possible to design more cost-effective and durable concrete mixtures. Ziolkowski P.’s 2023 study [54] explores the relationship between computational complexity and predictive accuracy in ML models applied to concrete mix design. The research evaluates five deep neural network models of varying complexity, trained on a dataset comprising concrete mix compositions and the corresponding compressive strength test results. The findings indicate a positive correlation between model complexity and predictive performance, as evidenced by improvements in the coefficient of determination (R^2^) and reductions in error metrics such as mean squared error (MSE) and root mean squared error (RMSE). By incorporating synthetic data generated through AI, the study enhances the robustness of model training and validation, ensuring greater reliability in strength prediction. Zhang, J. et al. [55] investigates the optimization of mix proportions and strength prediction of magnesium phosphate cement-based composites (MPCC) using ML models. The research employs an orthogonal experimental design with 32 mix proportions to examine the influence of various factors, such as MgO-to-phosphate ratio, water–binder ratio, aggregate–binder ratio, mineral admixture content, and steel fibre volume, on the flowability, setting time, and compressive strength of MPCC. The study finds that the MgO-to-phosphate ratio (M/P) has the most significant impact on early and long-term compressive strength, with a contribution of up to 83.6% at 28 days. The researchers further develop ML models, including random forest (RF) and the particle swarm optimization of backpropagation artificial neural networks (PSO-BP-ANN), to predict MPCC strength with high accuracy. The study highlights the potential of ML in optimizing mix designs and reducing experimental costs. These findings are particularly relevant for the development of high-performance, low-carbon construction materials. Golafshani, E. M. et al. [56] explore the application of ML techniques to optimize the compressive strength (CS), cost, and CO₂ emissions of recycled aggregate concrete (RAC) incorporating supplementary cementitious materials (SCMs). The authors compiled a dataset of 3519 samples from the literature and employed various ML algorithms, including Elastic Net regression, K-Nearest Neighbors, Artificial Neural Networks, and ensemble methods such as Random Forest, XGBoost, and CatBoost, to predict the CS of RAC. The best-performing model utilized a stacking approach, which improved RMSE by 4.2% over the next-best model. Sensitivity analysis using SHapley Additive Explanations (SHAP) and Monte Carlo simulations confirmed that concrete testing age and cement content significantly influence the CS. The study also introduced a multi-objective water cycle algorithm (MOWCA) to balance CS, cost, and sustainability in RAC mix designs. The results demonstrated that optimized mixes could achieve a CS between 37 MPa and 67.4 MPa while minimizing the cost and environmental impact. Additionally, a user-friendly graphical interface was developed to facilitate mix design optimization. The research aligns with sustainable construction trends by integrating AI-driven mix optimization for greener concrete production.

Recent advancements in ML for concrete mix design have highlighted the crucial role of both computational complexity and optimization algorithms in improving prediction accuracy. Computational complexity refers to the structure and depth of ML models, with more complex models being able to capture the intricate, nonlinear relationships between concrete components and their properties. However, increasing complexity can lead to challenges like overfitting, where the model performs well on training data but struggles with new, unseen data. Balancing this complexity to maintain generalizability is essential. Optimization algorithms are equally important because they adjust the model’s parameters during training to minimize prediction errors. Advanced optimization techniques like Adaptive Moment Estimation (ADAM) [57,58,59] and Stochastic Gradient Descent (SGD) [60,61,62] help models converge more efficiently and improve their performance on high-dimensional data. ADAM, for example, adapts learning rates for each parameter, improving the model’s ability to handle complex datasets, while SGD introduces randomness to help escape local minima and find better global solutions. In concrete mix design, where the data are often variable and include many factors, like mix ratios, additives, and environmental conditions, choosing the right optimization algorithm ensures that the model can accurately predict outcomes, such as compressive strength. As ML models become more complex to accommodate modern materials and intricate mix designs, the role of efficient optimization becomes even more critical. Together, computational complexity and optimization algorithms enhance the predictive power of ML models, enabling more accurate, efficient, and sustainable concrete mix designs.

## 3. Materials and Methods

### 3.1. Key Elements

ML models have demonstrated considerable effectiveness in predicting the technical properties of concrete, particularly compressive strength, based on the composition of the mix. This study aims to build on previous research [54] by not only examining the impact of computational complexity on the predictive performance of ML models but also investigating the influence of various optimization algorithms. Specifically, this research evaluates the performance of the Adaptive Moment Estimation (ADAM) algorithm and Stochastic Gradient Descent (SGD), alongside the previously utilized Quasi-Newton Method (QNM) [63,64,65]. The goal is to determine how these optimization methods interact with different levels of computational complexity to enhance the accuracy and efficiency of predicting concrete compressive strength. The depth of a Deep Neural Network (DNN) is a critical determinant of model complexity. Increasing the number of layers allows the model to capture more intricate patterns in the data, as each layer transforms the raw input into higher-level features [66,67]. While deeper networks offer the potential to model the complex relationships between concrete mix components, such as cement content, water, and admixtures, they also introduce challenges such as overfitting and vanishing gradients [68,69]. These issues become especially significant when the available training data are limited relative to the complexity of the model. Advanced optimization techniques, like ADAM and SGD, help mitigate these challenges by improving training efficiency and reducing the risk of overfitting. ADAM, in particular, adapts the learning rates for each parameter, making it more suitable for handling complex and sparse data, while SGD provides a stochasticity that helps escape local minima. The aim is to assess the impact of varying both the computational complexity (through changes in the number of layers and neurons) and optimization algorithms on the performance of the ML models. A total of forty-five DNN models were trained and tested, each with different configurations of layers and optimization techniques. The analysis aimed to understand how these factors interact to influence the predictive accuracy of compressive strength. The data used in this study were drawn from previous work [45,48,54] and include a comprehensive database of concrete mix recipes, along with results from corresponding compressive strength tests. This dataset, initially composed of several hundred records, was further expanded using a dedicated AI model to generate reliable synthetic data [70,71,72]. The expanded dataset allowed for a more robust evaluation of model performance across varying levels of complexity and optimization strategies. The concrete mix compositions included in the database were designed for a range of purposes and elements, incorporating variations in size and function, and the use of different admixtures. Given the diverse nature of the data, certain inconsistencies between mix designs, such as the presence of specific additives, were addressed through data preprocessing, including the removal of univariate outliers using a standard deviation-based method, as discussed later in the paper. The input variables for the models were the quantities of cement, the water–cement ratio, and the amount of fine aggregate and coarse aggregate, while the output variable was the compressive strength of the concrete samples. In line with other studies in the literature, this research focused on predicting compressive strength, a critical property for evaluating concrete performance. However, it is important to note that other factors, such as curing conditions [73,74] and the temperature at the time of concrete pouring [75], as well as other environmental exposures, also play significant roles in determining the overall quality of the concrete. While these factors were not the primary focus of this study, their inclusion in future research could provide a more comprehensive understanding of how to optimize concrete mix designs using ML. By examining both computational complexity and optimization algorithms, this study aims to identify strategies for developing more accurate and efficient AI-driven models for predicting concrete’s compressive strength. The results are expected to contribute valuable insights for advancing concrete technology, with a focus on increasing eco-friendliness, production efficiency, and predictive accuracy in concrete mix design.

### 3.2. Data Preparation and Processing

The dataset used in this research consists of 6187 records, which were generated using a dedicated AI model from an initial dataset of 741 records. These original data were derived from concrete recipes and compressive strength tests conducted under laboratory conditions following the PN-EN:206 standard [25]. Each record in the dataset includes several variables: f_ck_ (concrete compressive strength in MPa), C (cement in kg), WC (water-cement ratio), FA (fine aggregate in kg), and CA (coarse aggregate in kg). The parameters adopted for the study are shown in Table 1, and a fundamental statistical analysis of each variable was performed, including the maximum, minimum, mean, median, and mode values, which are presented in Table 2.

**Figure 1:**
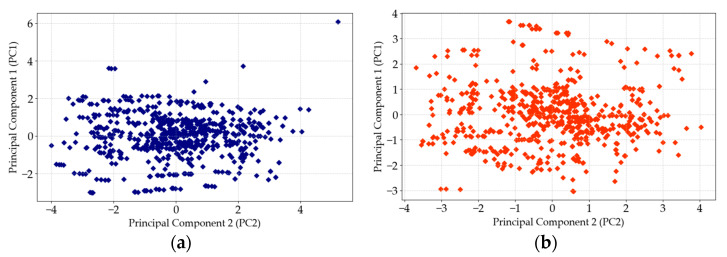
Principal Component Analysis: training data (**a**); synthetic data (**b**).

**Figure 2:**
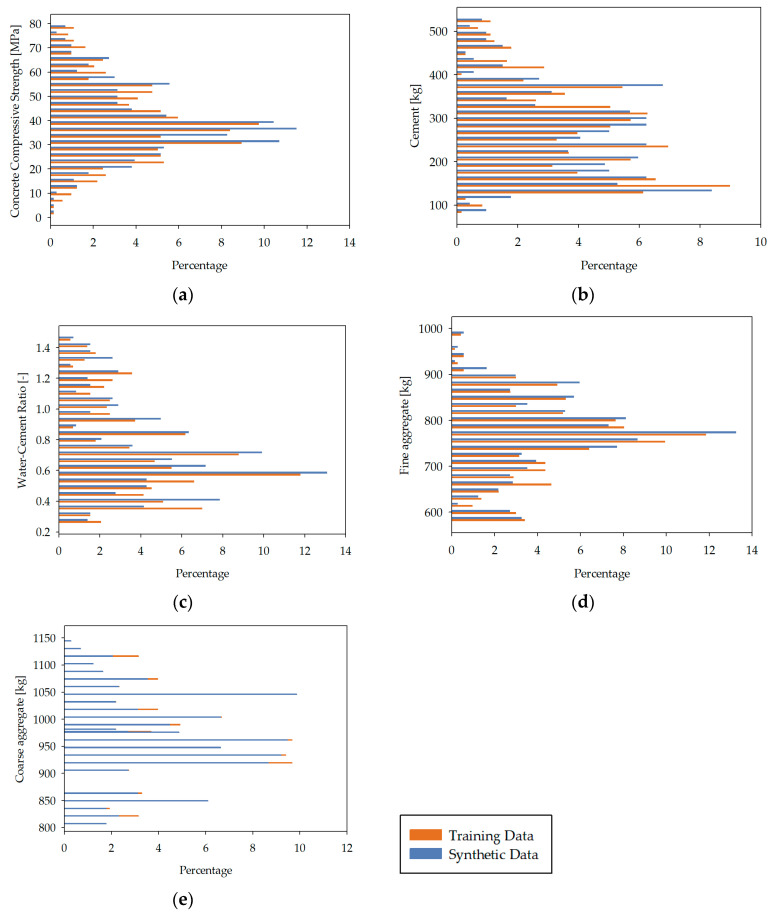
Field distribution comparisons: concrete compressive strength (**a**); cement (**b**); water–cement ratio (**c**); fine aggregate (**d**); coarse aggregate (**e**). Brown bars correspond to the training data. Blue bars correspond to synthetic data. Vertical axes are percentages [54].

To generate the synthetic data, the Tabular Long Short-Term Memory (TLSTM) model [76,77] was utilized, which allowed for the dataset to be significantly expanded while maintaining credible and reliable data quality. The quality of this synthetic data was verified through two key techniques: Principal Component Analysis (PCA) [78,79] and Jensen–Shannon Divergence (JSD) [80,81,82,83]. PCA was used to capture the essential structure of the dataset by identifying the most significant dimensions (Principal Components) of variability within the data, first for the original dataset and then for the synthetic dataset. The PCA evaluates whether the synthetic data conform to the structure encapsulated in the original data by comparing the proximity of the principal components. A synthetic quality score was calculated based on the distributional distance between the principal components of both datasets, with a closer alignment indicating higher quality. Figure 1 presents a visual comparison of the PCA results for the original and synthetic data. Furthermore, JSD was employed to compare the distribution of the original and synthetic data fields. The Jensen–Shannon Distance values between field distributions were calculated, where lower values indicate a closer resemblance and, therefore, higher-quality synthetic data. Visual comparisons of the field distributions for concrete compressive strength, cement, water–cement ratio, fine aggregate, and coarse aggregate are shown in Figure 2, where the orange bars represent the original data and the blue bars represent the synthetic data. Concrete compressive strength was evaluated in a laboratory setting using standardized samples in accordance with EN-206-1 [84]. The samples tested were cylindrical, with a diameter of 15 cm and a height of 30 cm, and cubic, with a side length of 15 cm. The results for the cubic samples were converted to their cylindrical equivalent strength, as required by the standard. The samples were made using ordinary Portland cement, with the maximum aggregate diameter not exceeding 20 mm, and clean, clay-free sand. The compressive strength tests were performed after 28 days, at which point the concrete had generally reached full strength. While the time required to achieve full strength may vary depending on the type of cement used, this study assumed that the cement did not significantly alter the time to the strength achievement. Any samples that had not reached full strength by 28 days were excluded from the dataset, ensuring consistency across all records. When training the artificial neural network (ANN) models, it is essential to operate within the maximum values of the input parameters to avoid extrapolating beyond the trained data, which could lead to unreliable predictions. The analysis presented in this study adheres to these boundaries, ensuring the integrity of the results.

**Figure 3:**
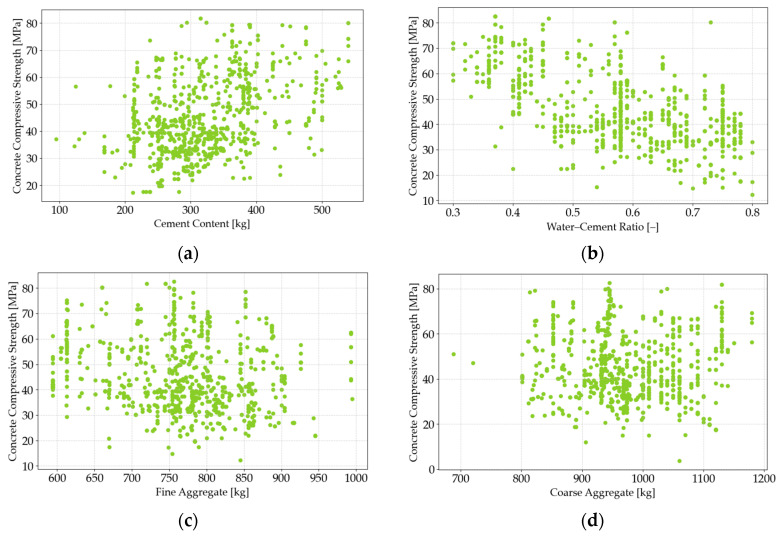
The scatter plots of target variable vs. input variables. The full compressive strength of concrete is on the vertical axis, expressed in MPa. The horizontal axis is the material content, expressed in kg for cement, fine-grained aggregate, and coarse-grained aggregate and L for water: cement (**a**); water–cement ratio (**b**), fine aggregate (sand 0–2 mm) (**c**), coarse aggregate (aggregate above 2 mm) (**d**).

To explore the relationship between input variables and the target variable (concrete compressive strength), scatter plots were generated. These visualizations provide a clear depiction of the proportional relationships between the variables, offering insights into how factors such as cement content, water–cement ratio, and aggregate composition affect compressive strength. Selected examples of these scatter plots are presented in Figure 3, illustrating the connection between the input variables and compressive strength. The target variable, compressive strength, is plotted on the vertical axis (in MPa), while the input variables (cement, water–cement ratio, fine aggregate, and coarse aggregate) are plotted on the horizontal axis (in kg or litres, depending on the variable). The use of scatter plots allows for a comprehensive visual analysis of variable interdependencies, which plays a crucial role in understanding how the components of a concrete mix contribute to the final strength. This approach enhances the predictive capacity of ML models by revealing key patterns that are central to optimizing concrete mix designs.

### 3.3. Model Training, Testing, and Selection Methodology

**Figure 4:**
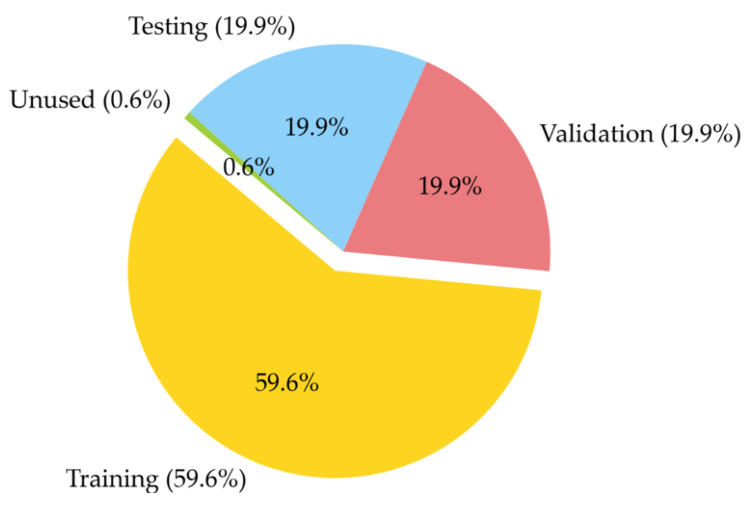
Pie chart of the division of subsets.

The database mentioned in Section 3.2 was used to train a series of deep artificial neural network models. The aim of this analysis was to investigate the impact of both computational complexity and optimization algorithms on the accuracy of predicted concrete compressive strength. The developed models, based on the quantitative composition of concrete mixtures, could estimate compressive strength. Five neural network models (MLM1–MLM5) were used, varying in the number of hidden layers, with each model trained using different optimization algorithms: the Quasi-Newton Method (QNM), Adaptive Moment Estimation (ADAM), and Stochastic Gradient Descent (SGD). This resulted in fifteen architectures in one series, and for validation purposes, each series was repeated three times (I, II, III), leading to a total of forty-five architectures in the entire experiment. In the series MLM1-MLM5, the computational complexity increases with the model number: MLM1 has two hidden layers, MLM2 has three, MLM3 has four, MLM4 has five, and MLM5 has six. The experiments were labelled as follows: as an example, ADAM-I-MLM2 indicates the Adaptive Moment Estimation (ADAM) optimization algorithm, a neural network architecture with three hidden layers in the first series. Each hidden layer in the neural network typically contains four neurons. The models used in this study were structured with five key parameters: four input variables and one output variable. To ensure efficient training of the deep neural networks, the dataset was divided into three independent subsets, training, validation (selection), and testing, following common practices in deep learning. The training set was employed to fine-tune the neural network’s parameters, while the validation set was used to monitor performance during the training process and to select the optimal model [85]. The test set, in turn, was used for the final evaluation of model performance. A procedure for identifying and removing outliers was applied, where any data points exceeding three times the median value of each variable were excluded. This method targeted univariate outliers to maintain the accuracy and reliability of statistical analyses. Outliers can significantly distort results, so their removal enhanced the representativeness of the data, contributing to more consistent and robust conclusions [86]. Although this process reduced the sample size, it was crucial for maintaining the integrity of the study. The final distribution of the dataset was as follows: 59.6% (3689 records) were allocated for training, 19.9% (1229 records) for validation, and 19.9% (1231 records) for testing, with 0.6% (38 records) left unused. A pie chart showing the presented subsets is depicted in Figure 4.

**Figure 5:**
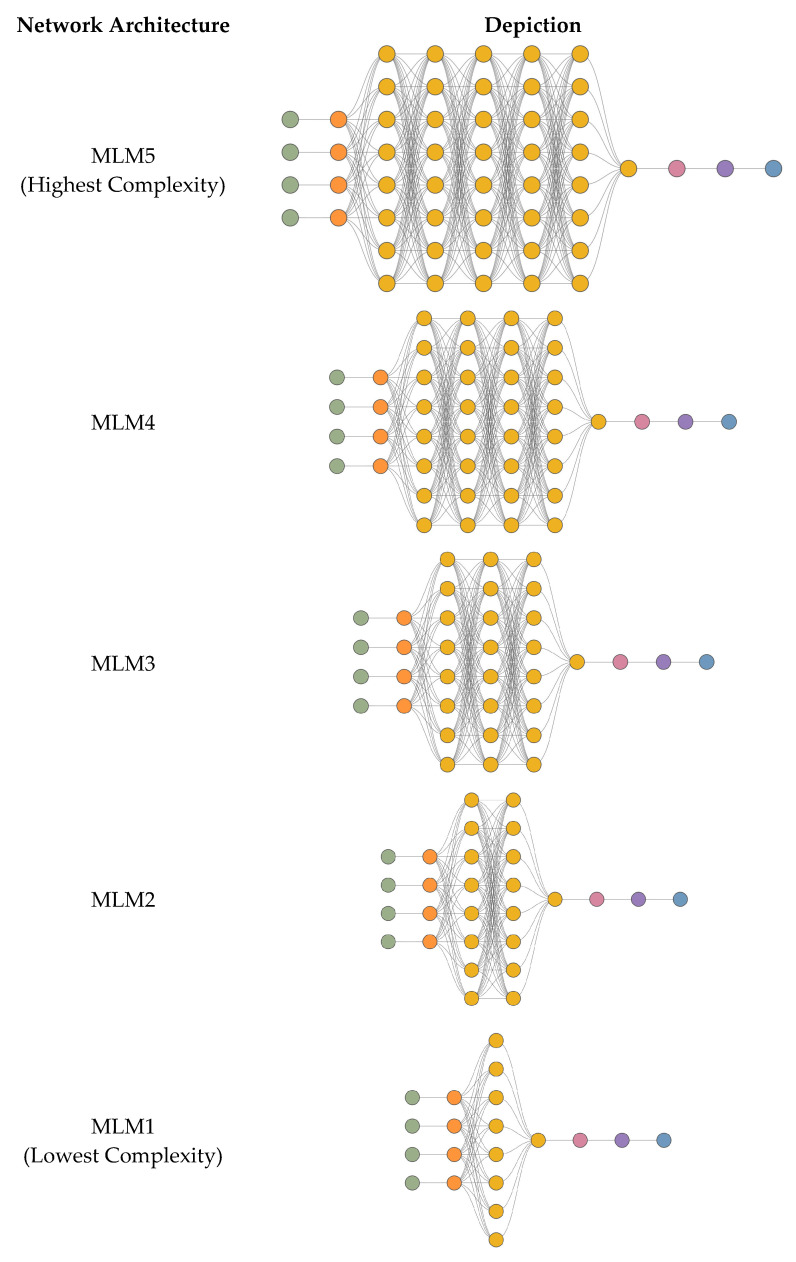
Deep neural network (DNN) topology for each model, from highest to lowest complexity of network architecture—input neurons (green), scaling neurons (orange), hidden neurons (yellow), descaling neurons (pink), bonding neurons (purple), and output neurons (blue).

The architecture of the first model (MLM1) was composed of 20 neurons. This includes four input neurons and nine neurons distributed across two hidden layers. Additionally, the model contains four neurons in a scaling layer, one neuron in a descaling layer, one neuron in a bonding layer, and one neuron in the output layer. The second model (MLM2) has a total of 28 neurons, with 4 input neurons and 17 neurons spread across three hidden layers, followed by a scaling layer with 4 neurons, a descaling layer, a bonding layer, and an output neuron. The third model (MLM3) comprises 36 neurons, including 4 input neurons and 25 neurons distributed over four hidden layers, along with 4 neurons in the scaling layer, 1 neuron in the descaling layer, 1 neuron in the bonding layer, and a single neuron in the output layer. The architecture of the fourth model (MLM4) contains 44 neurons: 4 input neurons, 33 neurons in five hidden layers, 4 neurons in the scaling layer, 1 neuron in the descaling layer, 1 neuron in the bonding layer, and 1 neuron in the output layer. Finally, the fifth model (MLM5) consists of 52 neurons, with 4 input neurons and 41 neurons distributed over six hidden layers, accompanied by 4 neurons in the scaling layer, 1 neuron in the descaling layer, 1 neuron in the bonding layer, and 1 output neuron. Figure 5 provides a visual representation of the architectures of the models used in the research.

The input variables, which represent the features of the data, were mapped to the input neurons of the neural network, while the output neuron was linked to the target variables. To improve the performance of the models, feature scaling was applied to all the datasets [87]. This involved transforming the numerical data into a standardized scale. The scaling and subsequent descaling were performed using the Mean Standard Deviation (MSD) technique. Throughout all models, consistent activation functions were employed: the hyperbolic tangent function for the hidden layers and a linear function for the output layer. Additionally, a bonding layer was incorporated into each network’s architecture. The models were carefully calibrated to minimize the associated loss function, with the error quantified using the Normalized Squared Error (NSE). Lower NSE values indicate stronger predictive abilities, while values approaching one suggest weaker performance. The closer the value is to zero, the better the model’s predictive accuracy. To further improve the model’s performance and prevent both overfitting and underfitting, regularization techniques were necessary. The L2 regularization method was selected for this purpose, as it minimizes the adjusted loss function, helping to reduce bias and thereby increase the precision of the predictions [88,89]. Regularization plays a crucial role in ensuring that the model captures meaningful patterns and relationships from the data without simply memorizing the training data. A well-regularized model can generalize effectively to new, unseen data. In particular, the L2 regularization method was highly effective, adding a penalty to the loss function based on the squared magnitude of the network parameters’ weights. This encourages smaller weight values, promoting the development of simpler models that are less prone to overfitting.

In this study, several optimization algorithms were utilized, including the Quasi-Newton Method (QNM), Adaptive Moment Estimation (ADAM), and Stochastic Gradient Descent (SGD). The QNM was one of the primary algorithms employed due to its well-established efficiency in addressing large-scale optimization problems. This method builds an approximation of the Hessian matrix by leveraging first-order derivative information, which allows it to handle second-order partial derivatives of the objective function. The QNM has been highly successful in solving nonlinear optimization problems across various fields, owing to its robustness and faster convergence compared to traditional gradient descent algorithms. Additionally, its ability to manage non-smooth functions makes it a versatile tool in optimization. Alongside QNM, the ADAM and SGD algorithms were also evaluated for their effectiveness in optimizing deep neural network parameters. ADAM, in particular, is known for its adaptive learning rate and efficient handling of sparse gradients, making it a powerful choice for training complex models. SGD, on the other hand, remains a classic but highly efficient optimization technique, especially in scenarios where computational resources are limited. Incorporating these algorithms allowed for a comprehensive comparison of their impact on the balance between computational complexity and convergence speed. The adopted training strategy, combining these algorithms, proved to be highly effective in optimizing model performance, achieving the desired level of accuracy while minimizing the computational resources required. It is important to emphasize that the models were trained using a specific dataset. Consequently, when applying these models, it is crucial to operate within the minimum and maximum value ranges specified in Table 2. This study does not take into account the influence of additives or admixtures on concrete properties. The practical range of the water–cement ratio considered in the dataset spans from approximately 0.3 to over 0.8. A ratio of 0.3 yields a very stiff mixture (unless superplasticizers are introduced), while a ratio of 0.8 results in a very wet and weak concrete. Any data points with a water–cement ratio outside this range were excluded from the dataset to ensure the integrity of the analysis.

## 4. Results and Analysis

In this study, five deep artificial neural network models (MLM1, MLM2, MLM3, MLM4, and MLM5), each with varying levels of computational complexity, were evaluated for their performance in predicting the compressive strength of concrete. The models ranged from the simplest, MLM1, to the most complex, MLM5, with an increasing number of hidden layers and neurons. The experiments were conducted in three series (I, II, and III), each utilizing different optimization algorithms: Quasi-Newton Method (QNM), Adaptive Moment Estimation (ADAM), and Stochastic Gradient Descent (SGD). The performance of each model was assessed by the coefficient of determination (R^2^), which indicates the model’s predictive accuracy. A higher R^2^ value reflects a better fit between the predicted and actual values, thereby demonstrating stronger model performance.

### 4.1. Data Processing Results

**Figure 6:**
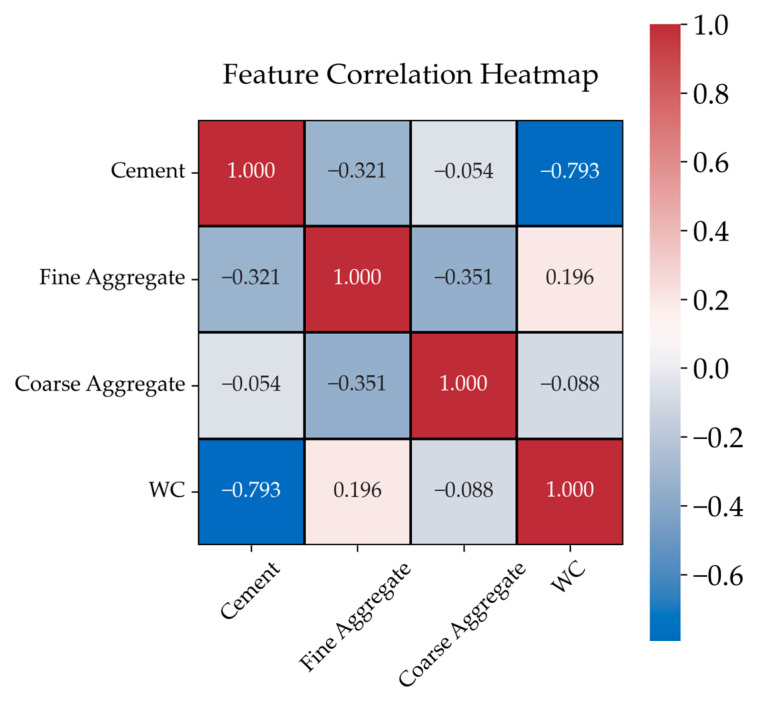
Feature correlation heatmap.

To better understand the relationships between the input variables and the output (compressive strength of concrete), a Feature Correlation Analysis was conducted. The results are presented in the form of a Feature Correlation Heatmap, as shown in Figure 6. This heatmap visualizes the degree of correlation between individual variables, where values closer to 1.0 indicate a stronger linear relationship. From the heatmap, it is evident that the water–cement ratio and cement content exhibit the strongest correlations with the output variable (compressive strength). These findings confirm that both variables play a critical role in determining the strength of concrete, which aligns with the findings in various studies. In particular, the water–cement ratio shows the highest correlation, highlighting its significant influence on concrete’s performance. Other input variables, such as the amount of fine aggregate, also show a moderate correlation with the output variable, with a more notable impact compared to water content. Interestingly, the water content itself has a stronger effect than the amount of coarse aggregate, further emphasizing the importance of the balance between water and cement in the mix. These insights align with existing literature, where the water–cement ratio and cement content have been frequently identified as the key determinants of concrete’s compressive strength. The relationships highlighted in the Feature Correlation Heatmap offer a deeper understanding of how different mix components interact to influence the final strength of concrete, and they serve as a foundation for further optimization of the models used in this study.

**Figure 7:**
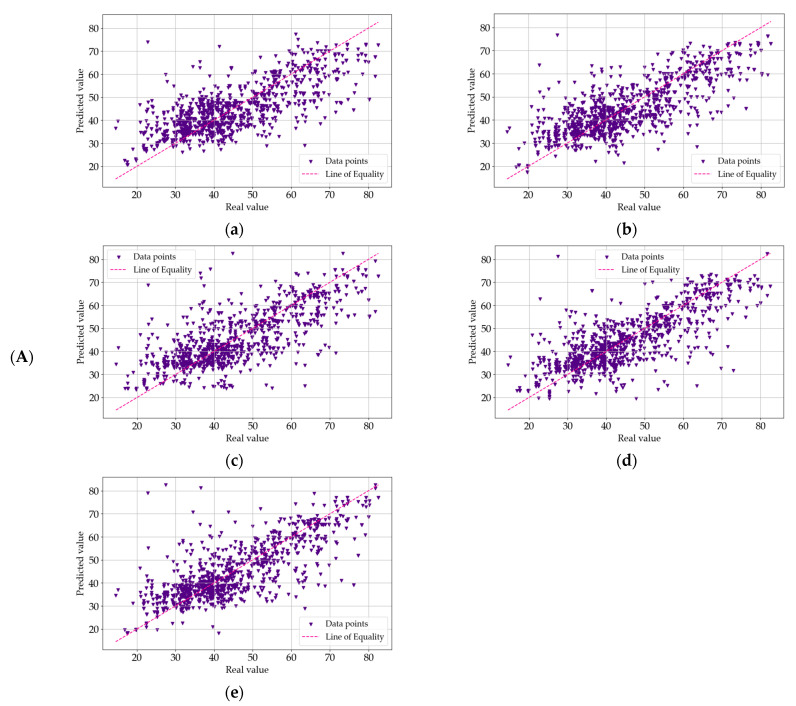

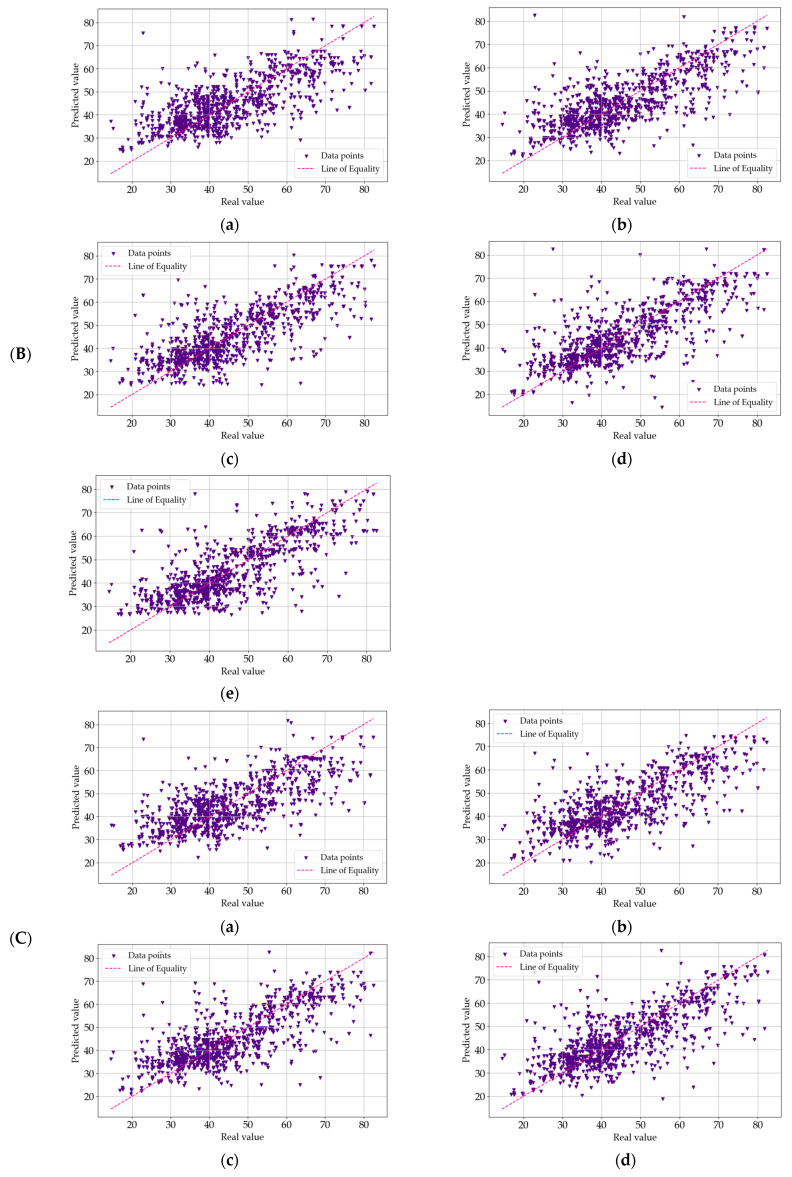

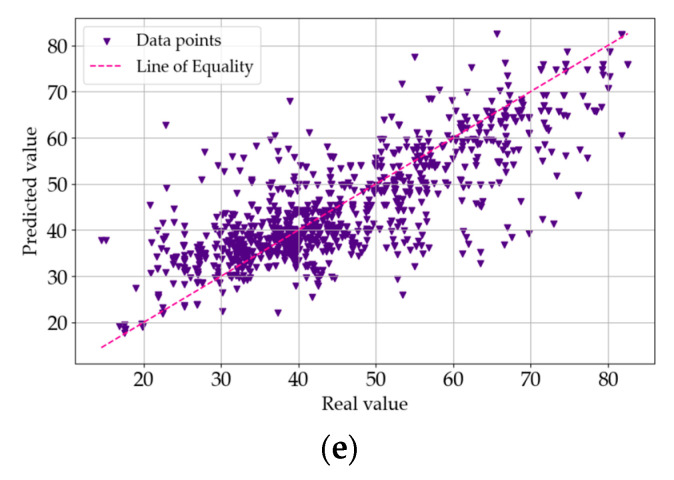
(**A**) Goodness-of-fit chart for MLM1 (**a**), MLM2 (**b**), MLM3 (**c**), MLM4 (**d**), and MLM5 (**e**) in series I—optimization algorithm using the Quasi-Newton Method (QNM). Shows the predicted value of the target variable versus the real one. (**B**) Goodness-of-fit chart for MLM1 (**a**), MLM2 (**b**), MLM3 (**c**), MLM4 (**d**), and MLM5 (**e**) in series II—optimization algorithm using the Quasi-Newton Method (QNM). Shows the predicted value of the target variable versus the real one. (**C**) Goodness-of-fit chart for MLM1 (**a**), MLM2 (**b**), MLM3 (**c**), MLM4 (**d**), and MLM5 (**e**) in series III—optimization algorithm using the Quasi-Newton Method (QNM). Shows the predicted value of the target variable versus the real one.

**Figure 8:**
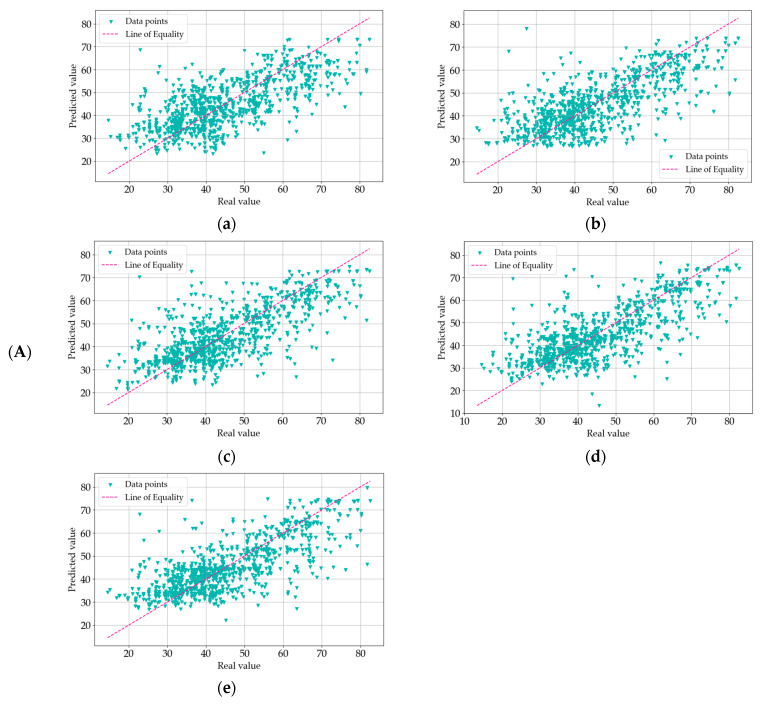

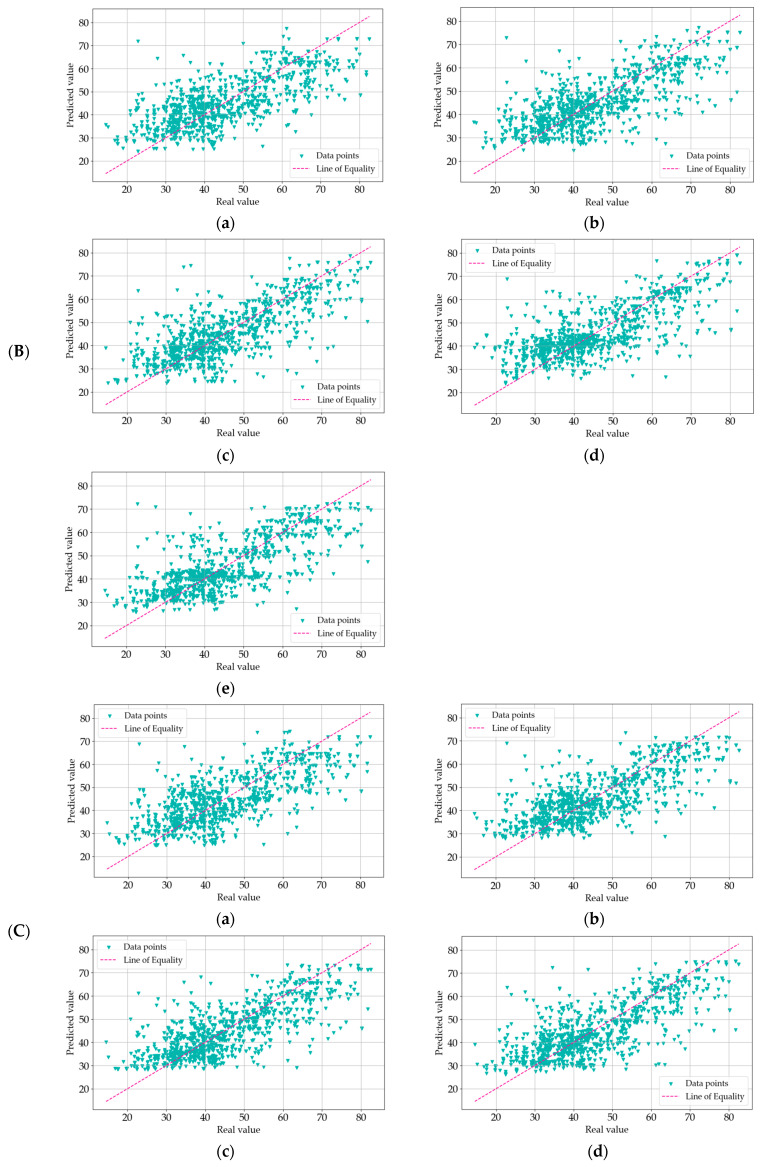

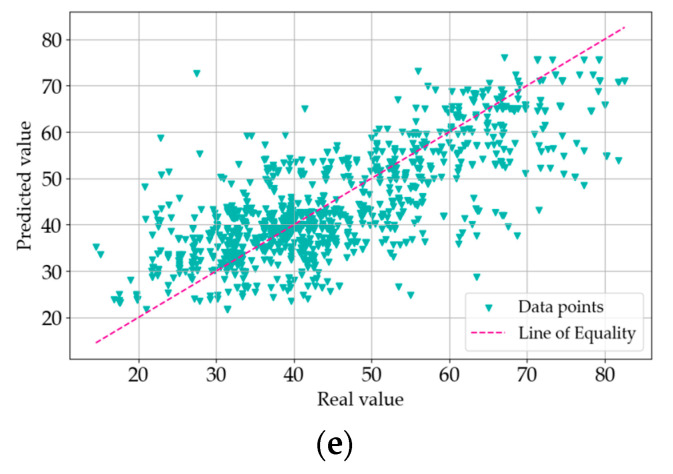
(**A**) Goodness-of-fit chart for MLM1 (**a**), MLM2 (**b**), MLM3 (**c**), MLM4 (**d**), and MLM5 (**e**) in series I—optimization algorithm using Adaptive Moment Estimation (ADAM). Shows the predicted value of the target variable versus the real one. (**B**) Goodness-of-fit chart for MLM1 (**a**), MLM2 (**b**), MLM3 (**c**), MLM4 (**d**), and MLM5 (**e**) in series II—optimization algorithm using Adaptive Moment Estimation (ADAM). Shows the predicted value of the target variable versus the real one. (**C**) Goodness-of-fit chart for MLM1 (**a**), MLM2 (**b**), MLM3 (**c**), MLM4 (**d**), and MLM5 (**e**) in series III—optimization algorithm using Adaptive Moment Estimation (ADAM). Shows the predicted value of the target variable versus the real one.

**Figure 9:**
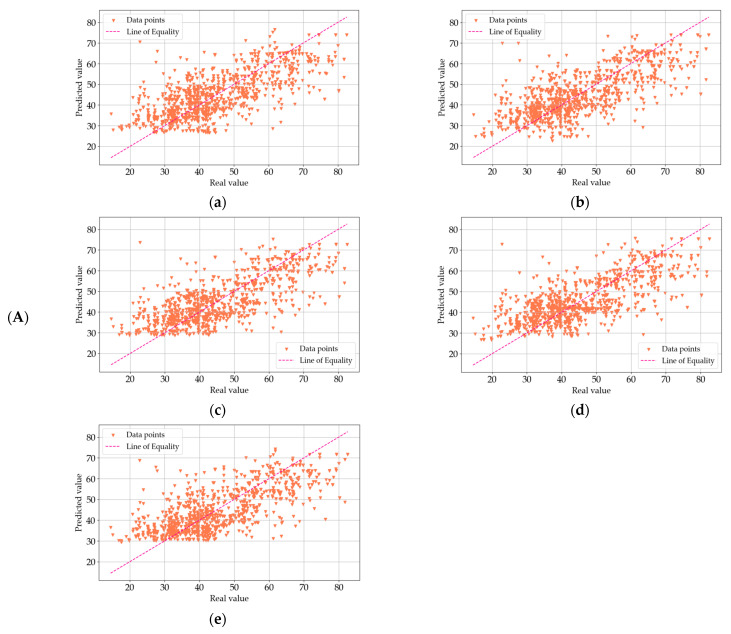

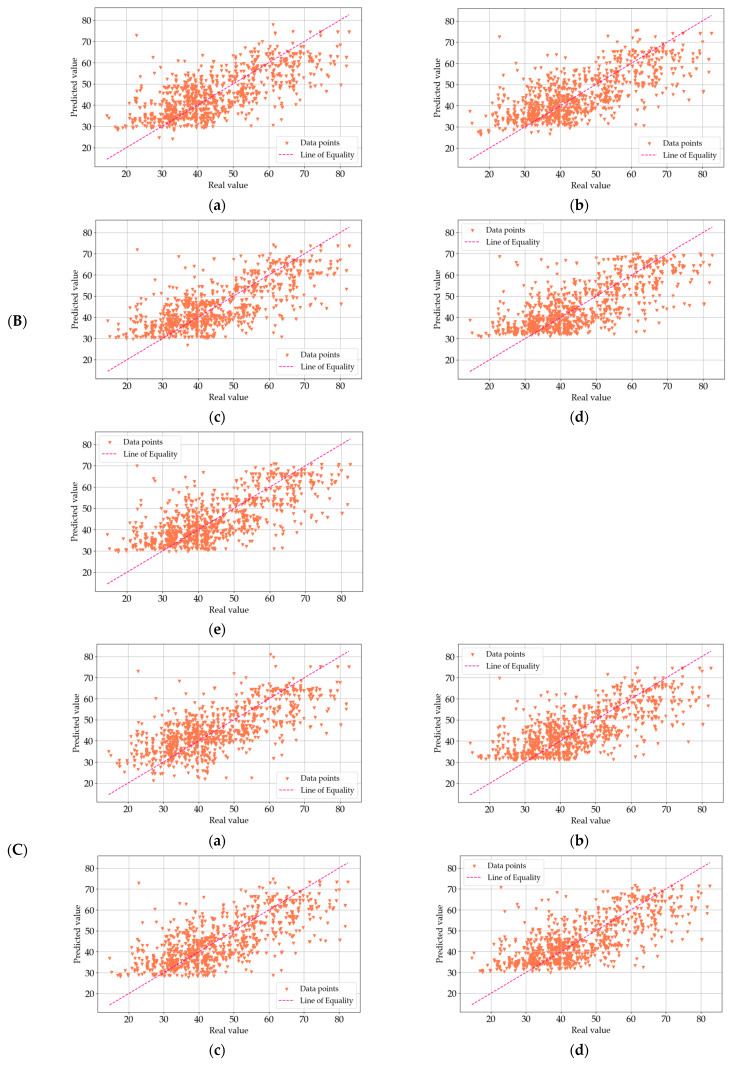

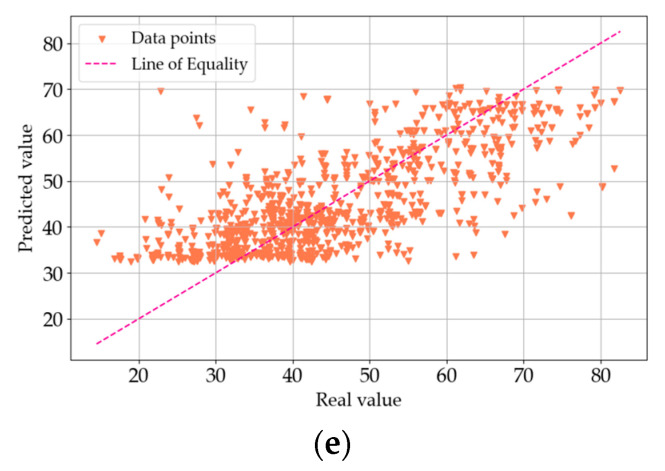
(**A**) Goodness-of-fit chart for MLM1 (**a**), MLM2 (**b**), MLM3 (**c**), MLM4 (**d**), and MLM5 (**e**) in series I—optimization algorithm using Stochastic Gradient Descent (SGD). Shows the predicted value of the target variable versus the real one. (**B**) Goodness-of-fit chart for MLM1 (**a**), MLM2 (**b**), MLM3 (**c**), MLM4 (**d**), and MLM5 (**e**) in series II—optimization algorithm using the Stochastic Gradient Descent (SGD). Shows the predicted value of the target variable versus the real one. (**C**) Goodness-of-fit chart for MLM1 (**a**), MLM2 (**b**), MLM3 (**c**), MLM4 (**d**), and MLM5 (**e**) in series III—optimization algorithm using Stochastic Gradient Descent (SGD). Shows the predicted value of the target variable versus the real one.

Each of the models in this study was subjected to a comprehensive goodness-of-fit test. This assessment provided a quantitative measure of the discrepancy between the observed (actual) values and those predicted by the models. A commonly used metric for evaluating the goodness-of-fit in scientific research is the coefficient of determination, denoted as R^2^. This parameter is pivotal in determining the proportion of variation in the output variable that is explained by the model. In essence, the R^2^ value quantifies how well the predicted values from the model correspond to the actual data points. An R^2^ value of 1.0 signifies a perfect fit, where the model’s predictions perfectly align with the target values. On the other hand, lower R^2^ values indicate a greater degree of discrepancy between the predicted and observed values. Figure 7, Figure 8 and Figure 9 provide a visual representation of the goodness-of-fit analysis for the three series (I—a, II—b, and III—c), using the R^2^ coefficient as the primary measure. These figures illustrate the performance of each model across different optimization algorithms, highlighting the correlation between model complexity, optimization method, and predictive accuracy. Through this analysis, it is evident that more complex models, such as MLM4 and MLM5, achieved higher R^2^ values, especially when optimized using the ADAM algorithm. The goodness-of-fit test confirms that these models were better suited for predicting the compressive strength of concrete, as they accounted for a larger fraction of the variation in the output data.

**Figure 10:**
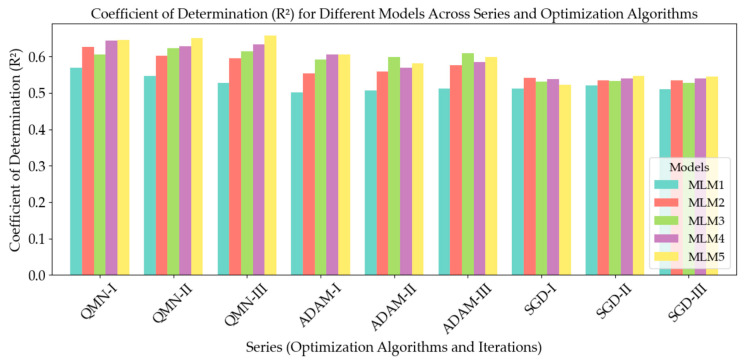
The coefficient of determination (R^2^) values for MLM1, MLM2, MLM3, MLM4, and MLM5 in series I, II, and III.

In the graphs presented in Figure 7, Figure 8 and Figure 9, the model exhibits relatively higher discrepancies between the predicted and measured compressive strengths when the values exceed 60 MPa or fall below 30 MPa. A key reason for this trend lies in the dataset’s composition: there are notably fewer samples within these extreme ranges, which reduces the model’s ability to learn representative patterns for very high- or very low-strength concretes. Consequently, when the model encounters test points in these underrepresented regions, the predictions are prone to larger errors. To mitigate this, we propose two primary avenues for future improvement. The first is expanding the training data to include more samples, either through additional experimental campaigns or external databases, that specifically target very high- and low-strength concretes and employing data augmentation or incorporating domain-specific knowledge (e.g., the known effects of certain admixtures on high-strength or low-strength mixes) to enrich the model’s understanding of such extreme cases. By bolstering the data density in these under-sampled regions, the predictive robustness of our models across the entire strength spectrum is expected to improve significantly. The first optimization method we applied was the Quasi-Newton Method (QNM). In series I, the R^2^ values were 0.5691 for MLM1, 0.6268 for MLM2, 0.6053 for MLM3, 0.6438 for MLM4, and 0.6453 for MLM5. This trend continues with series II, where the values were slightly lower, with 0.5467 for MLM1, 0.6017 for MLM2, 0.6227 for MLM3, 0.6285 for MLM4, and 0.6514 for MLM5. In III, the R^2^ values slightly decreased again, yielding 0.5272 for MLM1, 0.5959 for MLM2, 0.6136 for MLM3, 0.6337 for MLM4, and 0.6571 for MLM5. These results suggest a consistent series improvement in predictive accuracy as model complexity increased, with MLM5 consistently outperforming simpler models. Next, the Adaptive Moment Estimation (ADAM) algorithm was employed. In series I, the R^2^ values were 0.5020 for MLM1, 0.5540 for MLM2, 0.5912 for MLM3, 0.6059 for MLM4, and 0.6052 for MLM5. Series II showed similar values, with 0.5062 for MLM1, 0.5591 for MLM2, 0.5991 for MLM3, 0.5696 for MLM4, and 0.5805 for MLM5. Series III produced slightly better results, with R^2^ values of 0.5114 for MLM1, 0.5753 for MLM2, 0.6093 for MLM3, 0.5843 for MLM4, and 0.5992 for MLM5. ADAM showed a relatively stable performance across series, although it did not reach the highest R^2^ values achieved by QNM. Lastly, the Stochastic Gradient Descent (SGD) algorithm was tested. In series I, the R^2^ values were 0.5115 for MLM1, 0.5415 for MLM2, 0.5313 for MLM3, 0.5386 for MLM4, and 0.5231 for MLM5. Series II saw marginal improvements, with 0.5207 for MLM1, 0.5337 for MLM2, 0.5332 for MLM3, 0.5405 for MLM4, and 0.5463 for MLM5. Series III, however, revealed some consistency, with values of 0.5100 for MLM1, 0.5349 for MLM2, 0.5278 for MLM3, 0.5401 for MLM4, and 0.5446 for MLM5. Compared to ADAM and QNM, SGD showed lower overall R^2^ values, particularly for the more complex models. The results reveal that the more complex models (MLM4 and MLM5) consistently yielded better R^2^ values across all optimization methods and series, demonstrating stronger predictive accuracy. QNM emerged as the most effective optimization method in this study, particularly for the higher-complexity models. These findings underscore the importance of both model complexity and the choice of optimization algorithm in enhancing the predictive power of ML models in concrete compressive strength prediction. The detailed R^2^ values for each model, optimization method, and series are presented in Figure 10, offering a comprehensive overview of model performance across the experiment.

### 4.2. Analysis of R^2^ Trends

**Figure 11:**
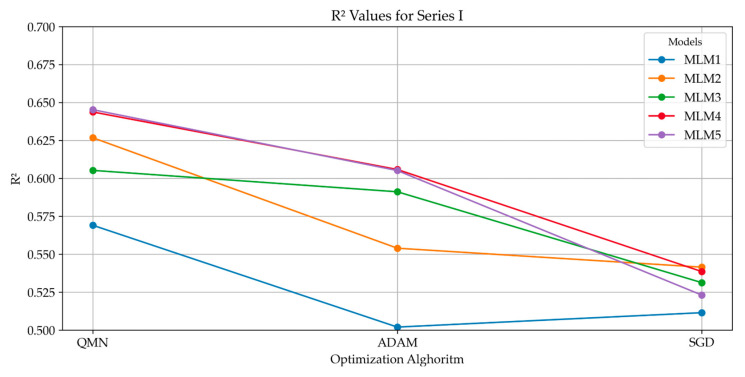

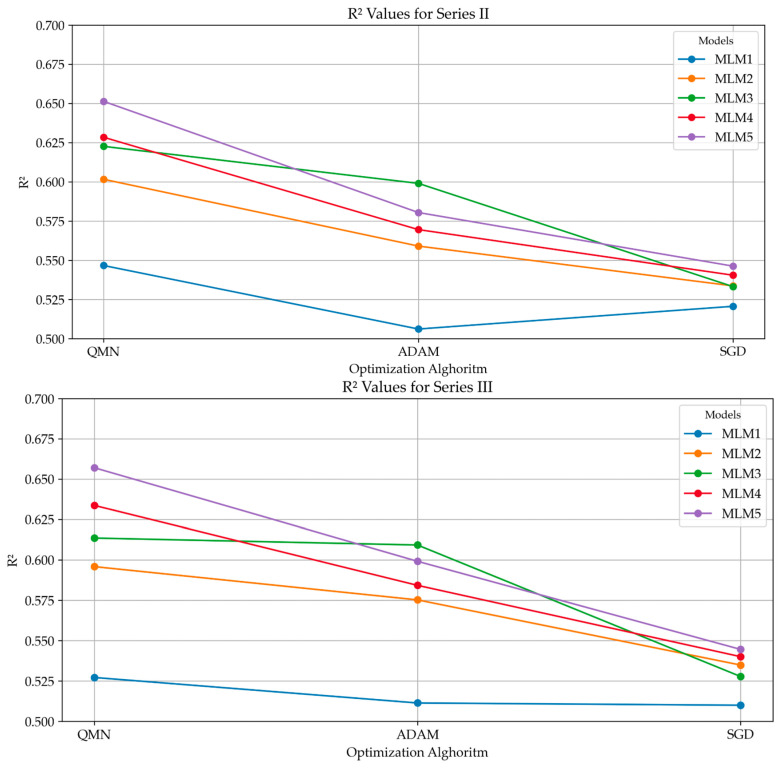
R^2^ values for different models across optimization methods and series.

The R^2^ values calculated for each model, optimization method, and series provide critical insights into the performance of the deep neural networks in predicting the compressive strength of concrete, as presented in Figure 11. These trends reveal important relationships between model complexity, the choice of optimization algorithm, and the overall predictive accuracy. The results consistently indicate that increasing the complexity of the neural network models improves their predictive accuracy. Across all optimization methods and series, the more complex models (MLM4 and MLM5) yielded higher R^2^ values compared to the simpler models (MLM1 and MLM2). This suggests that models with more hidden layers and neurons are better able to capture the underlying relationships in the data, leading to more accurate predictions of compressive strength. However, the gains in R^2^ between MLM4 and MLM5, while present, are relatively small, suggesting that further increasing model complexity beyond MLM4 offers diminishing returns. Thus, there appears to be a threshold in complexity beyond which the improvement in predictive accuracy is marginal. This finding implies that while more complex models are preferable, practical considerations such as computational cost and efficiency must be weighed against the relatively modest accuracy gains. The choice of optimization algorithm significantly affected the performance of the models. Among the three algorithms tested, the Quasi-Newton Method (QNM), Adaptive Moment Estimation (ADAM), and Stochastic Gradient Descent (SGD). QNM consistently produced the highest R^2^ values, particularly for the more complex models. This demonstrates that QNM is well-suited for optimizing deep neural networks in the context of concrete compressive strength prediction, likely due to its ability to efficiently converge on solutions even in highly complex models. In contrast, ADAM exhibited lower R^2^ values across models and series compared to QNM, though its performance was more stable. ADAM’s adaptive learning rate allows for reliable convergence across different datasets, but the algorithm may not always achieve the highest possible accuracy in this specific context. However, its robustness across series makes it a viable option when stability is prioritized over maximal predictive power. SGD, while a widely used optimization method, showed the weakest performance in this study. The R^2^ values for models optimized using SGD were consistently lower across all series, particularly for the more complex models. This suggests that SGD may be less effective for optimizing deep neural networks in this domain, likely due to its slower convergence and sensitivity to hyperparameters. The performance of the models also varied across the three series. For models optimized using QNM, a slight decrease in R^2^ values was observed from series I to series III. This suggests that while QNM is effective, the predictive accuracy of the models may vary in different iterations. Despite this, the overall performance remained strong, particularly for the more complex models. In contrast, ADAM demonstrated greater stability across series, with less variation in R^2^ values. This highlights ADAM’s robustness in handling data variability, making it a reliable choice for applications where stability is crucial. However, the lower R^2^ values compared to QNM suggest that this stability comes at the cost of reduced predictive accuracy. SGD exhibited the most consistent R^2^ values across series, but the lower overall performance suggests that while it offers stability, it lacks the ability to optimize models for high predictive accuracy in this application.

Consistently higher R^2^ values were obtained with the Quasi-Newton Method (QNM) compared to Stochastic Gradient Descent (SGD). This outcome can be attributed to QNM’s use of approximate Hessian information (second-order derivatives), which allows for optimization to proceed more efficiently in deep models with complex loss landscapes. In contrast, SGD is more susceptible to becoming trapped in local minima or saddle points, particularly when the learning rate and batch sizes are not carefully tuned. As a result, a stronger predictive performance was observed with QNM.

### 4.3. Analysis of Model Errors

**Figure 12:**
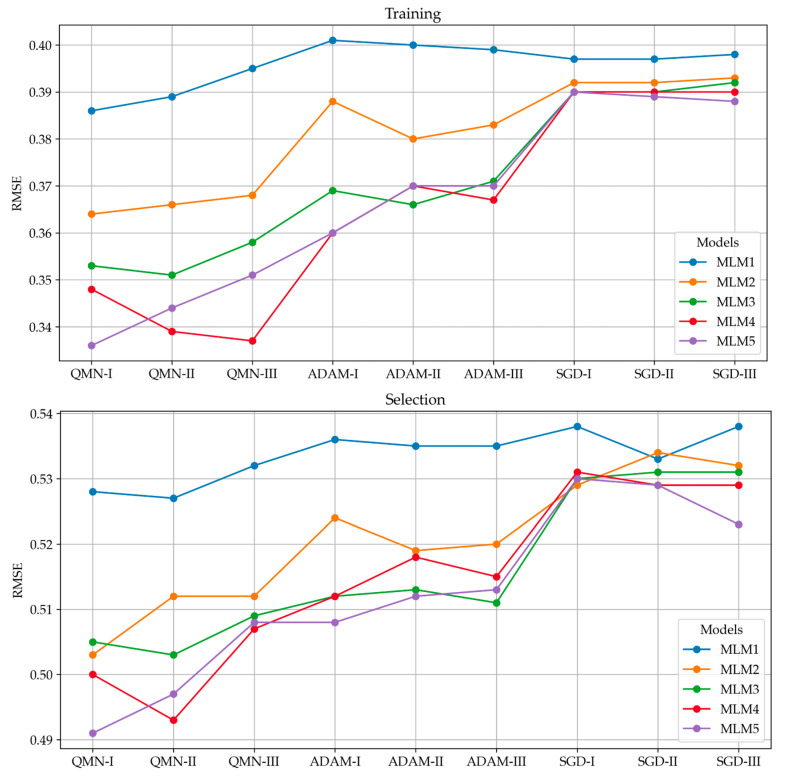

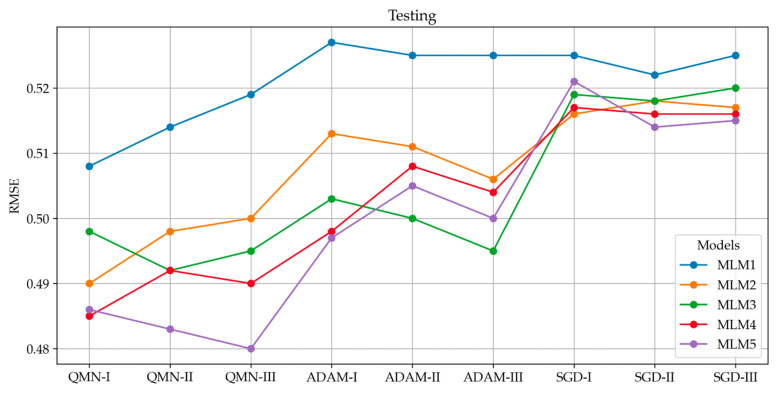
Training, selection, and testing of root mean squared error (RMSE) values for MLM1–MLM5 in series I–III.

Error analysis was conducted to rigorously evaluate the performance of each model across all series and optimization methods. This analysis involved calculating a range of error metrics, including Sum Squared Error (SSE), Mean Squared Error (MSE), Root Mean Squared Error (RMSE), Normalized Squared Error (NSE), and Minkowski Error (ME). These metrics provide a comprehensive view of the model’s accuracy by quantifying the discrepancies between the predicted and actual values for concrete compressive strength. The error analysis revealed important insights into the consistency and precision of the models [90,91]. In addition to the global error metrics, a detailed report was generated, outlining the maximum and minimum values, as well as the mean and standard deviation for absolute, relative, and percentage errors with respect to the test data. These statistics offer a more granular view of model performance, allowing for a deeper understanding of where the models excel or struggle in terms of prediction accuracy. Histograms were constructed for the test subsets, illustrating the distribution of errors across predictions. These histograms provide a tangible representation of error spread, highlighting whether the models tend to overpredict or underpredict compressive strength values and by how much. The error distributions also aid in identifying patterns, such as skewness or the presence of outliers, which could be targeted for further refinement in future model iterations [92]. The comprehensive error evaluation performed in this study not only assesses the overall performance of the models but also reveals potential weaknesses that could be addressed in future work, like models with larger standard deviations in error may benefit from further regularization or more robust feature engineering [93]. Figure 12 presents the RMSE metric for each model, categorized by series and optimization method; other metrics, such as SSE, MSE, NSE, and ME, presented similar patterns. This breakdown allows for a clear comparison of model performance and optimization efficacy, highlighting the impact of different algorithms on error reduction.

**Figure 13:**
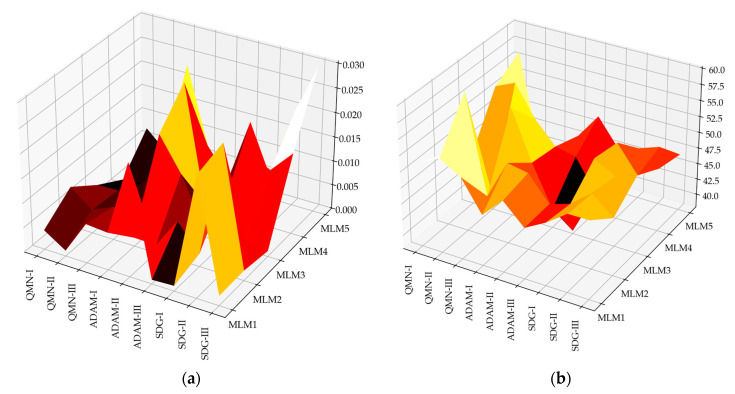

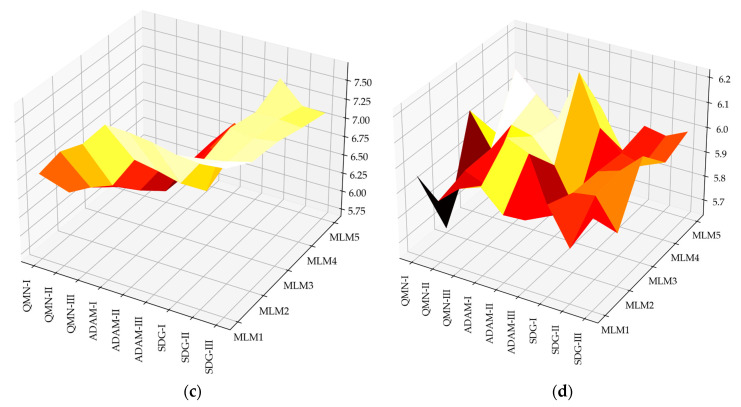
The values of absolute error [minimum (**a**), maximum (**b**), mean (**c**), deviation (**d**)] for MLM1–MLM5 across different optimization algorithms and series.

A clear error downward trend can be noticed for the RMSE metric in the Training, Selection, and Testing sections in all three series, with an increase in computational complexity. The trend suggests that higher computational complexity allows for better learning and generalization, contributing to more precise predictions of concrete compressive strength. Statistical calculations were conducted for individual target variables, as presented in Figure 13. Figure 13 shows the minimums, maximums, averages, and standard deviations in the absolute error of the model for the test data. Calculations of relative error and percentage error were also conducted and showed similar patterns.

It can be observed that the average error decreases with the increase in the model’s computational complexity for absolute error. This trend suggests that models with a greater number of hidden layers and neurons exhibit improved predictive accuracy, reducing the discrepancy between predicted and actual values. The reduction in error is particularly noticeable in more complex models, such as MLM4 and MLM5, which consistently demonstrate lower mean absolute errors across different optimization methods. This indicates that deeper architectures allow the network to capture more intricate relationships within the dataset, leading to enhanced generalization and reduced variance in predictions. This improvement in accuracy is observed across various error metrics, reinforcing the significance of model complexity in mitigating both systematic and random errors. However, while increasing complexity generally results in better performance, the diminishing returns beyond a certain threshold highlight the need to balance model depth with computational efficiency. Although AI-based methods demonstrate significant potential in predicting concrete compressive strength based on mix composition, they are not without limitations. One prominent drawback is the risk of overfitting, especially in deeper architectures with a greater number of hidden layers and neurons. Overfitting occurs when a model learns not only the underlying patterns in the training data but also its noise and idiosyncrasies, resulting in poor generalization to unseen datasets. This issue is particularly relevant when the training dataset is limited in size or diversity, as the model may fail to account for the full range of variability in real-world concrete production scenarios. To mitigate this, techniques such as regularization, cross-validation, and data augmentation are often employed, though they add complexity to the model development process. Another critical limitation is the dependence of ML-based methods on large, high-quality databases. The predictive accuracy of these models hinges on the availability of comprehensive datasets that capture a wide array of mix compositions, environmental conditions, and production variables. In practice, collecting such extensive data can be resource-intensive and challenging, particularly when considering the influence of factors like curing conditions, weather impacts, and aggregate characteristics, which were not explicitly modelled in this analysis. Incomplete or biassed datasets can skew model predictions, limiting their reliability in scenarios where data are sparse or unrepresentative of the target application. This dependency underscores the need for robust data collection frameworks to support the practical deployment of ML in concrete mix design. Furthermore, the lack of interpretability in certain deep ML models poses a significant challenge, particularly for engineers and practitioners, who require actionable insights from predictive tools. While complex models like MLM4 and MLM5 excel at capturing intricate relationships within the data, their ‘black-box’ nature often obscures the reasoning behind specific predictions. Unlike simpler regression-based approaches, where the influence of individual variables can be directly assessed, deep architectures with numerous layers and parameters offer limited transparency. This lack of interpretability can hinder trust in AI-based predictions and complicate efforts to refine mix designs based on model outputs. Techniques such as feature importance analysis or surrogate models can partially address this issue, but they introduce additional layers of approximation that may not fully resolve the underlying opacity. While the presented models focus on predicting concrete’s compressive strength based on its mix composition, it is important to recognize that various additional factors, particularly those linked to environmental conditions and production processes, also significantly influence concrete strength. One major consideration is the appropriate curing of concrete immediately after placement. Inadequate curing can severely degrade its properties, especially long-term durability. Another pressing concern is placing concrete under adverse weather conditions, such as early freezing during hydration or excessive drying in hot climates, both of which can compromise the material’s performance. Environmental aggression likewise poses notable risks to concrete quality, such as carbonation [94], and chloride attack in coastal settings can adversely affect durability. Moreover, additives and admixtures are commonly used in concrete to enhance the production process and are integral to achieving the desired properties. The shape, texture, and origin of aggregates also affect their workability and durability, although they tend to have a more profound impact on fresh concrete than on its hardened state [95]. In addition, the gradation and size distribution of aggregates dictate the necessary volume of paste needed for adequate workability [96]. In this analysis, certain variables, namely the specifics of the technological process, environmental conditions, and raw material characteristics, were not explicitly considered, as it was assumed that the quality of the sampled concrete met an acceptable standard.

## 5. Summary and Conclusions

This study examined how computational complexity and different optimization algorithms influence the predictive performance of deep neural network (DNN) models when estimating the compressive strength of concrete. Five models (MLM1–MLM5), progressively increasing in depth and neuron count, were trained using the Quasi-Newton Method (QNM), Adaptive Moment Estimation (ADAM), and Stochastic Gradient Descent (SGD) algorithms. A comprehensive dataset was employed, combining experimental laboratory measurements of concrete properties with an augmented set of synthetic records generated through a Tabular Long Short-Term Memory (TLSTM) procedure. The findings demonstrate that increases in computational complexity generally enhance the models’ predictive accuracy, although these marginal benefits tend to diminish when moving to a different architecture, such as moving from MLM4 to MLM5. QNM proved to be the most effective optimization method in terms of achieving high coefficients of determination (R^2^), especially for deeper networks, whereas ADAM displayed stable yet slightly lower accuracy, and SGD converged more slowly and underperformed in the prediction metrics. An in-depth error analysis, encompassing the Sum Squared Error, Mean Squared Error, Root Mean Squared Error, Normalized Squared Error, and Minkowski Error, corroborated these results, showing that deeper architectures trained with more advanced optimization strategies produced lower and more tightly distributed errors across the test samples. The crucial role of the water–cement ratio and cement content was reaffirmed, in agreement with the established findings in concrete technology, while the specific impact of fine aggregate also emerged as a factor warranting further attention. Despite the demonstrated potential of ML in predicting concrete compressive strength, certain constraints must be acknowledged. The models concentrated primarily on mix composition and did not incorporate critical external variables such as curing regimes, climatic conditions during pouring, and the influence of various chemical admixtures and additives. Although the diversity of compositions in the dataset mitigates some of these limitations, ensuring that all predictions lie within the boundaries of the training set remains essential to maintain model validity. Expanding the scope of these models and refining their accuracy will require a more inclusive approach. Incorporating detailed information on the curing process, environmental parameters, and a broader range of aggregate characteristics could capture a fuller picture of real-world conditions. Future work may also examine the interplay between sustainability criteria, such as reducing CO_2_ emissions through cement substitutions or advanced admixtures, and a broader range of concrete properties, including tensile strength, shrinkage, creep, and frost resistance, beyond just compressive strength. While the current models focus on compressive strength, single-property optimization may overlook trade-offs that affect overall performance. Consequently, developing AI-based models for multi-objective optimization that account for these interdependencies could offer a more holistic approach, ensuring that both sustainability and durability are addressed. Achieving this would require larger, more diverse datasets capturing additional properties, a critical step toward enhancing the practical applicability of such models. Building on this, future research should also investigate the impact of synthetic data on model generalizability in more detail. Validation strategies, such as t-SNE visualizations or ablation studies, would help to evaluate the potential performance differences between models trained with and without synthetic data. Addressing the computational cost implications, particularly for large-scale real-world deployments, remains crucial. A comparative analyses of training times and resource utilization across various architectures could inform practitioners of the trade-offs between model complexity and practical feasibility. In parallel wit this, exploring ensemble learning approaches or physics-informed neural networks that incorporate fundamental principles of cement hydration and microstructure development may further refine model accuracy. Embedding interpretability methods, especially those aligned with explainable artificial intelligence, could illuminate how different input variables are weighed, offering transparent guidance to engineers for adjusting mix designs. Region-specific datasets and transfer learning methods could be especially valuable when tailoring models to local materials and environmental conditions. Additionally, incorporating hyperparameter tuning through metaheuristic or hybrid optimization strategies, as well as real-time analytics from on-site sensors, may facilitate adaptive systems capable of dynamically adjusting mix proportions based on instantaneous feedback. In such scenarios, high-performance computing (HPC) environments could expedite training and inference, enabling more immediate recommendations for large-scale construction projects. This research highlights the potential of deep learning in tackling the complexity of modern concrete design. Robust optimization methods and deeper architectures jointly enhance accuracy, offering a credible and efficient alternative to traditional empirical, iterative approaches. By integrating more comprehensive datasets, real-world operational variables, and advanced optimization techniques, AI-driven mix design is poised to become an indispensable tool that supports both performance and sustainability goals in contemporary construction.

## Figures and Tables

**Table 1:** The parameters adopted in the dataset [54].

Parameter	Compressive Strength	Cement	Water–Cement Ratio	Sand 0–2 mm	Aggregate Above 2 mm
Type	Target	Input	Input	Input	Input
Description	The 28-day compressive strength of concrete that is considered to have most of its strength (MPa).	Content of cement added to the mixture, expressed in (kg/m^3^).	Water-to-cement ratio (−).	Content of fine-grained aggregate added to the mixture, expressed in (kg/m^3^).	Content of coarse-grained aggregate with a size more than 2 mm, added to the mixture, expressed in (kg/m^3^).

**Table 2:** Value ranges of database input variables [54].

Input Variable	Minimum	Maximum	Mean	Median	Dominant
Cement	87.00 kg/m^3^	540.00 kg/m^3^	322.15 kg/m^3^	312.45 kg/m^3^	380.00 kg/m^3^
Water–cement ratio	0.30	0.80	0.58	0.58	0.58
Fine-grained aggregate (sand 0–2 mm)	472.00 kg/m^3^	995.60 kg/m^3^	767.96 kg/m^3^	774.00 kg/m^3^	594.00 kg/m^3^
Coarse aggregate (aggregate above 2 mm)	687.80 kg/m^3^	1198.00 kg/m^3^	969.92 kg/m^3^	963.00 kg/m^3^	932.00 kg/m^3^

## Data Availability

The original contributions presented in the study are included in the article, further inquiries can be directed to the author.
